# Nontrivial nonradiating all-dielectric anapole

**DOI:** 10.1038/s41598-017-01127-2

**Published:** 2017-04-21

**Authors:** Nikita A. Nemkov, Ivan V. Stenishchev, Alexey A. Basharin

**Affiliations:** 1 National University of Science and Technology “MISiS”, The Laboratory of Superconducting metamaterials, 119049 Moscow, Russia; 2 Moscow Institute of Physics and Technology (MIPT), 141700 Dolgoprudny, Moscow region Russia; 3 National University of Science and Technology “MISiS”, Department of Theoretical Physics and Quantum Technologies, 119049 Moscow, Russia; 4 Institut Langevin, CNRS UMR 7587, ESPCI Paris, PSL Research University, 1 rue Jussieu, 75005 Paris, France

## Abstract

Dynamic anapole is a promising element for future nonradiating devices, such as cloaked sources and sensors, quantum emitters, and especially the sources for observing dynamic Aharonov-Bohm effect. However, the anapole response can be damped by the Joule losses. In this paper we theoretically propose and experimentally demonstrate a novel type of active all-dielectric source, which is in some sense, realizes the elementary anapole of Afanasiev, and study its radiative/nonradiative regimes in the microwave range.

## Introduction

Static toroidal moment, also known as the *static anapole* (from the Greek. An - neg. particle and polos - pole) is a peculiar type of source which confines magnetic fields within its domain of definition. In 1957 Zel’dovich introduced the anapole concept to explain the parity violation of the weak interactions in atomic nuclei^1^. Toroidal moments are now recognized as a separate multiple family which complements electric and magnetic ones^2–4^. A simple representative, the toroidal dipole moment, corresponds to the poloidal currents on the surface of a torus and can be pictured as a wire solenoid bent into a torus shape. Zel’dovich proposed that such excitation can be present in the atomic nuclei due to the static currents. Since the prediction of the static toroidal dipole its significance has been discussed in a number of solid-state systems, including ferroelectrics, nano-ferro- magnetics, multiferroics, molecular magnets and *etc*.^5–12^.

Dynamic toroidal dipole can also be defined in terms of the the poloidal torus currents, but now time-dependent. Being physically different from the dynamic electric dipole moment in current configuration, the source defined by the dynamic toroidal dipole moment radiates with the same angular momentum and the far-field properties. Therefore, the toroidal and electric dipole moments are indistinguishable for any distant observer^13–16^. However, the electric and magnetic spherical multipole coefficients are complete and not associated with the current origin of the source in near fields. But, the current Cartesian multipole expansion gives us possibilities to calculate separately the toroidal and electric dipoles contributions required the currents in near-field zone of the source^15, 16^. This peculiar property is responsible for the fact that the toroidal family stayed unrecognized for so long, as well as points out its relevance for many problems in photonics^15, 16^. It should be noted that the toroidal dynamic concept is very close to an idea contained in van Bladel book^17^, where the some term is called as ***P***_***e2***_ component in multipole expansion (See formulas 7.150 and 7.151), but describes exactly a toroidal moment. Furthermore, combining the toroidal and electric dipoles in such a way that they interfere destructively in far-field zone one obtains a simple example of a nontrivial nonradiating source known as *dynamic anapole*. Importantly, the current origin of them are different in near-field zone.

Dynamic anapole is thus characterized by a superposition of the electric **P** and toroidal **T** dipole moments which are in a specific relation, namely **P** = *ik* **T** ^13, 15^. Note that in the static case *k* = 0 and the electric moment disappears **P** = 0 making the static anapole synonymous with the static toroidal dipole. As shown in ref. 18 the point dynamic anapole may be viewed as the basic building block out of which an arbitrary nonradiating source can be composed. Dynamic anapole modes are relevant to many phenomena in electrodynamics and beyond. For instance, their existence proves that the inverse scattering problem of the electrodynamics is unsolved without additional assumptions about the source structure^19^. Moreover, given the obvious toroidal topology of a large number of biologically important molecules and protein complexes, we expect that the study of electromagnetic interactions associated with the excitation of toroidal and anapole modes can explain many processes in nature^20–23^.

Despite the long theoretical history experimental observation of the dynamic toroidal response has become possible only recently in connection with the progress in metamaterials. These artificially structured on the subwavelength scale media provide an opportunity to access new and exotic optical phenomena^24–30^. For the first time dynamic toroidal dipole response was demonstrated in 2010^31^ in metamaterials consisting of specifically designed metallic metamolecules of the toroidal topology. These metamolecules featured suppression of the electric and magnetic dipole moments, while toroidal one was spectrally isolated and resonantly enhanced to the measurable level. This demonstration opened the way for verification of amazing phenomena of toroidal electrodynamics and stimulated the studies of metamaterials and plasmonic systems exhibiting strong toroidal response in 3D, planar and dielectric metamolecules^15, 16, 31–42^.

A previous study investigated metamolecules consisting of four closely spaced dielectric microcylinders of the lithium tantalate, which thereby provide a near-field coupling between Mie-magnetic modes excited in each cylinder^35^. Exploring the toroidal topology of dielectric cluster, the authors were able to observe an unusual configuration of electromagnetic fields in the metamolecules, in which the electric and toroidal moments, excited in a metamolecule interfered destructively in far field zone and hence formed an anapole. As result, the metamolecules exhibited suppressed radiating losses.

Remarkably, a metamaterial consisting of cylindrical dielectric clusters, a priori, has low dielectric losses. Due to the anapole excitations the scattering fields do not undergo the radiation losses, and due to the dielectric ingredients avoid Joule losses. Thus, the metamaterial becomes transparent for the observer at the anapole frequency. Obviously, anapole can emerge in other all-dielectric structures^15, 16, 35–41^.

Nontrivial nonradiating source confines electromagnetic fields to its volume of support. For a very small nonradiating source this amounts to highly concentrated fields. There are no fundamental limitations on this concentration and the fields of an idealized point anapole correspond to δ- functions^13, 18^. Due to the strongly concentrated fields in a small volume anapole acts as a perfect resonator with an arbitrarily high Q-factor^43^. Of course, in real systems there are restrictions liming the Q-factor.

As a particularly interesting application of the dynamic anapole we mention the possibility of their use for the time-dependent Aharonov-Bohm experiment^34, 44^. As explained in ref. 18, although there are additional complications in the time-dependent case the anapole provide a promising ground both for theoretical and experimental study of the time-dependent Aharonov-Bohm effect. One of the necessary ingredients is a non-radiating source with a particular topology and controllable properties. In the present paper we demonstrate at the first time an experimental design of a nontrivial nonradiating all-dielectric anapole source which in some sense realizes the elementary anapole of Afanasiev *et al*. and study its properties in details. The proposed source can be viewed as an elementary ingredient for more complicated nonradiating sources^18^, and provides possibilities to explore the dynamic Aharonov-Bohm effect, active quantum emitters, antennas for secure communications and invisibility problems.

## Results

### The structure of the system

Dynamic anapole consists of toroidal and electric dipoles. There are many approaches available for experimental implementation of the electric dipole. However, forming the toroidal dipole is a currently developing problem. We note that fabrication of 3D metamolecules with toroidal topology is quite challenging^15^. In contrast, the metamolecules formed as clusters of dielectric particles are technologically preferable, especially in the THz and visible optics ranges.

**Figure 1:**
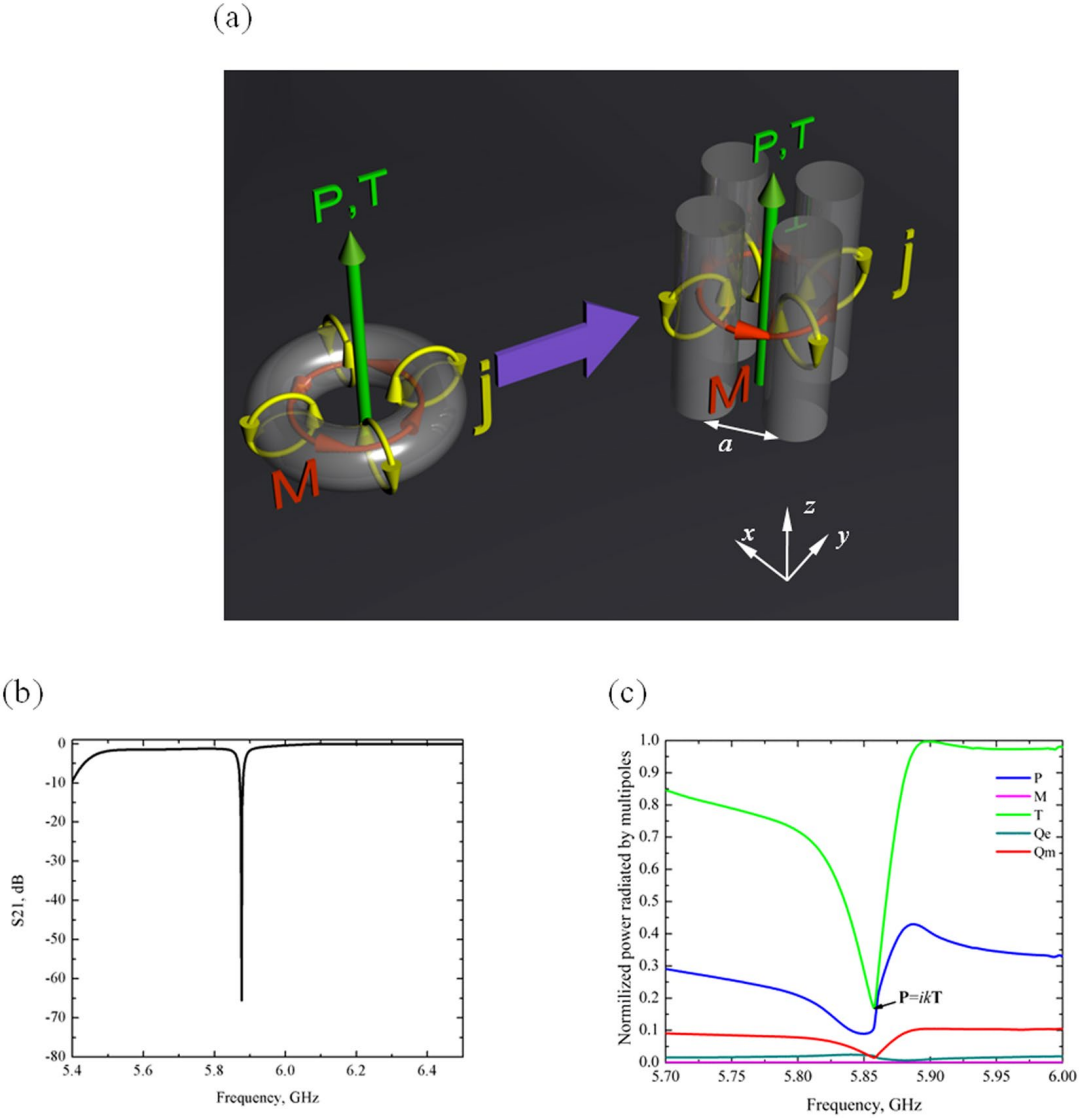
(**a**) Representative toroidal source and an all-dielectric source supporting anapole excitation. Its metamolecule is composed of four closely spaced infinitely long high-index dielectric cylinders. Yellow arrows show displacement currents ***j*** induced by the vertically polarized electric dipole antenna (**P**), red arrows show magnetic moments **M** of the constituent dielectric cylinders, and the green arrow represents the electric field **E**, toroidal and electric moments. (**b**) The calculated spectrum S21 of power received at second antenna relative to the power input to the anapole source composed of four cylinders, as shown in Fig. 1a. The results correspond to the HFSS simulations. (**c**) Contributions of the five strongest multipole excitations to the radiation of the anapole source.

We propose a simple way to realize the toroidal dipole in a system of four dielectric cylinders placed in the corners of a square. At the frequency of the magnetic Mie-resonance mode each cylinder acquires a magnetic moment **M** resulting from the displacement currents **j**, oscillating in each cylinder. These moments are aligned head-to-tail and form a vortex of magnetic field within a metamolecule^35^ (Fig. 1a). The similarity between this mode and the dynamic toroidal dipole is presented on the Fig. 1a. For such configuration the total magnetic dipole and quadrupole moments are vanishing (this can be seen via symmetry) so that the toroidal dipole moment is the leading term in the multipole expansion^3^. Indeed, a point toroidal dipole corresponds to the following current

1 $${{\boldsymbol{j}}}_{toroidal}=c\,{{\rm{curl}}}^{2}{\bf{T}}{{\rm{\delta }}}^{(3)}({\bf{r}})$$

while the point magnetic dipole is produced by

2 $${{\boldsymbol{j}}}_{magnetic}=c\,{\rm{curl}}\,{\bf{M}}\,{{\rm{\delta }}}^{(3)}({\bf{r}})$$

Now, the four magnetic dipoles of value **M** placed at the sides of a square and directed along them correspond to the following current density

3 $$\begin{array}{rcl}{{j}}_{4magnetic}(r) & = & {\bf{M}}\,{\rm{curl}}\,\hat{{\bf{y}}}\,{\rm{\delta }}({\bf{r}}-\hat{{\bf{x}}}a)-{\bf{M}}\,{\rm{curl}}\,\hat{{\bf{x}}}\,{\rm{\delta }}({\bf{r}}-\hat{{\bf{y}}}a)\\  &  & -{\bf{M}}\,{\rm{curl}}\,\hat{{\bf{y}}}\,{\rm{\delta }}({\bf{r}}+{\bf{x}}a)+{\bf{M}}\,{\rm{curl}}\,\hat{{\bf{x}}}\,{\rm{\delta }}({\bf{r}}+\hat{{\bf{y}}}a)\end{array}$$

Here we assume that the magnetic moments **M** are located in the *x*, *y*- plane with unit basis vectors $\hat{{\bf{x}}},\hat{{\bf{y}}},\hat{{\bf{z}}}$ and *a* is equal to half of the square side. Expanding this expression to the leading order in *a/r* one gets

4 $${{\boldsymbol{j}}}_{4magnetic}(r)=2a{\bf{M}}\,{\rm{curl}}(\hat{{\bf{x}}}{\nabla }_{{\rm{y}}}-\hat{{\boldsymbol{y}}}{\nabla }_{{\rm{x}}})\delta ({\bf{r}})+O({a}^{2}/{r}^{2})$$

Hence, to the leading order in *a/r*, this combination of magnetic dipoles forms a toroidal dipole of the magnitude T = *4a* *M* pointing in the *z* direction, i.e. orthogonal to the plane of the magnetic dipoles. Adding the electric dipole with **P** = *ik* **T** to the center of the cluster, one obtains the dynamic anapole.

### The results of simulations

We now propose an all-dielectric metamolecule that is free of dissipation and capable of supporting the anapole mode in the microwave regime. The metamolecule is based on four high-index dielectric cylinders excited by an electric dipole antenna placed at the center between cylinders. The dipole is characterized by the electric dipole moment **P** (depicted as **P** on Fig. 1a). We note that this metamolecule is active in the sense that it is excited internally alike antenna by feeder cable, in contrast to scattering metamolecules which respond to the excitation by an external plane wave. Thus, the electromagnetic response of the metamolecule is underpinned by the displacement currents **j**, which are induced in each cylinder by the electric dipole antenna **P**. This configuration creates dynamically induced and spatially confined magnetization circulating along a loop **H**, thereby promoting near-field coupling between the Mie-type magnetic moments **M** excited in each cylinder. At specific frequency these moments become aligned head to tail, forming a dynamic vortex of magnetic field. This configuration represents toroidal dipole **T** destructively combined with electric dipole **P** and forms a dynamic anapole, due to destructive interference between their radiations in far-field zone. Hence, in this way an anapole mode is dynamically generated and one expects that the electromagnetic fields will be confined to the near-field zone and strongly suppressed away from the metamolecule. Since ideal nonradiating anapole do not exist (due to reciprocity theorem), we mean “nonradiating” to compensation of leading multipoles excited in our source. However, higher multipoles exist in the system as quadrupoles, octupoles, with low radiating power^16^.

The cylinders of radius *R* = 5 mm and height 35 mm are placed with the center-to-center separation *a* = 10 mm. They are made of SrTiO_3_, a dielectric ceramics that is known to exhibit a strong response at the microwave frequencies. The dielectric permittivity of SrTiO_3_ is rather high reaching ε ~ 37. The cylinders are surrounded by vacuum.

The source is excited by the electric dipole **P** feeding by a standard coaxial port (see Fig. 1a). The electromagnetic properties of the source are computed with the aid of a commercial Maxwell’s equation solver HFSS using the standard modeling approach. The results of simulations reveal the anapole response around frequency 5.85 GHz. The first strong indication manifests as a resonance dip in the S21 spectrum. Here, S21 represents the power received at the second antenna placed at the distance 4λ from the source, in far-field zone (see Fig. 1b) relative to the power input to the anapole source^45^. At the frequency 5.85 GHz the S21 is less than −60 dB which means negligible received signal at port 2.

The next step to examine is the multipole expansion of the system with account for the toroidal moments. The multipole moments are calculated based on the density of the conductive and displacement currents present in the dipole and the cylinders^14^. The results of the multipole expansion are presented in Fig. 1c, which indicates the radiation power conditioned by the five leading multipoles: toroidal **T**, electric **P**, and magnetic **M** dipoles as well as electric **Q**_**e**_ and magnetic **Q**_**m**_ quadrupoles. At the frequency 5.85 GHz the radiation powers of the toroidal **T** and electric **P** dipoles indeed coincide as required by the structure of the anapole mode. All the other multipoles are strongly suppressed, by orders of magnitude in comparison with the toroidal and electric dipoles. We thus conclude that the high Q-factor of resonance at 5.85 GHz is attributed to the anapole excitation, i.e. the interference between the electric and toroidal dipoles. Previously, the high Q-factor response was discussed in 3D, planar toroidal and anapole metamaterials^32–34, 43, 46^.

**Figure 2:**
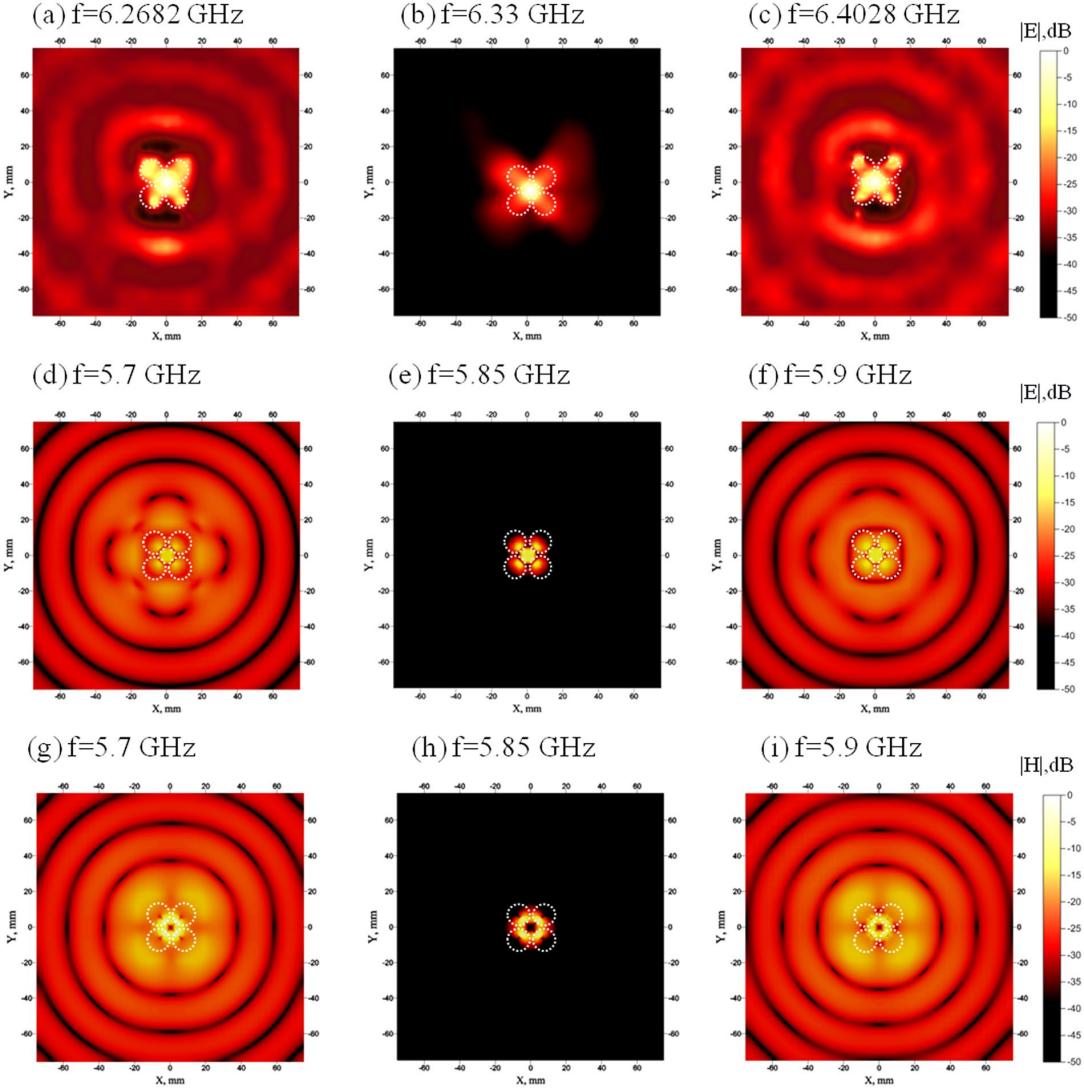
(**a**–**c**) Measured electric field distributions on the top of the source normalized on maximum field scanned by the 2D probe on the frequencies 6.2682 GHz (toroidal mode), 6.33 GHz (anapole mode) and 6.4028 GHz (toroidal mode), respectively. (**d**–**f**) Simulated electric field distributions on the top of the source normalized on maximum field on the frequencies 5.7 GHz (toroidal mode), 5.85 GHz (anapole mode), 5.9 GHz (toroidal mode), respectively. (**g**–**i**) Magnetic field distributions at the same frequencies as (**d**–**f**).

**Figure 3:**
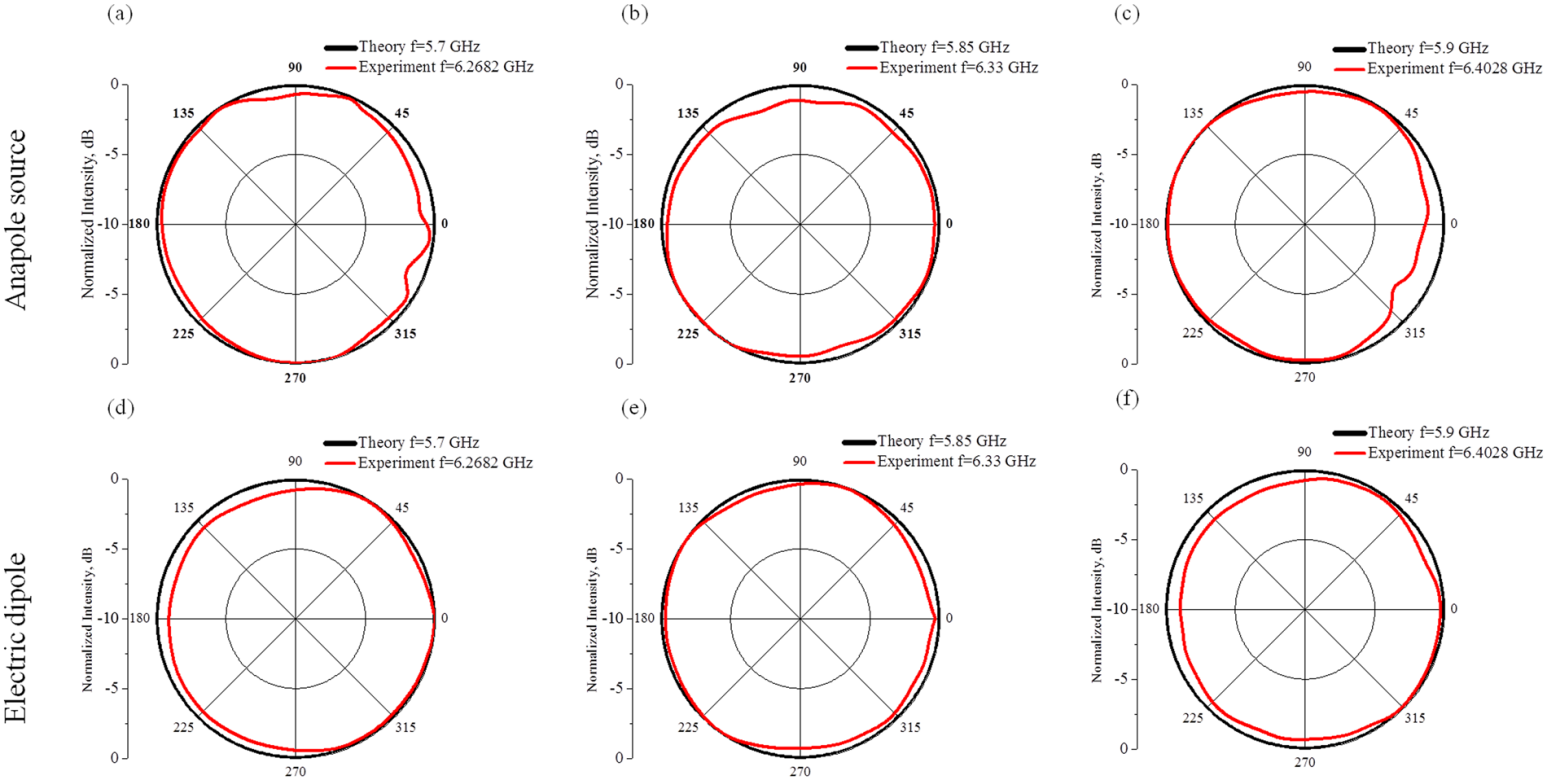
Normalized radiation Patterns (RP) of Anapole source (**a**–**c**) and Electric dipole, for comparison (**d**–**f**). Black curve represents results of simulation and red curve is experimental results.

We also plot a detailed map of the electric and magnetic fields distributions on top of the source (see Fig. 2e,h). As Fig. 3b demonstrates the normalized radiation pattern (RP) of the system is almost omnidirectional in *x*, *y* plane at the anapole frequency. Hence the radiation is absent in any direction and not just towards the position of the second antenna. During the paper, we plot the radiation patterns as relative amplitudes, normalized to amplitude on the antenna boresight (the maximum of radiation intensity). Thereby, the “0” dB shows the maximum of normalized radiation^45^. One sees that the fields are virtually absent in the far-field zone and constitute less than −50 dB in amplitude. At the same time, in the near-zone fields behave according to the theoretical expectations. The magnetic field **H** (Fig. 2h) forms a distinct vortex that penetrates through all the cylinders, while being absent in the center of the metamolecule. The electric field distribution reaches its maximum in the central region (Fig. 2e).

Also, we would like to emphasize the following point. On the frequencies adjacent to the anapole dip the source is radiating. According to the multipole expansion (Fig. 1c), at these frequencies the toroidal dipole moment dominates other contributions. However, the radiation at the frequencies f = 5.7 GHz and f = 5.9 GHz is very similar to the electric dipole radiation with the same RP (Fig. 3a–c). This is yet another indication of the indistinguishability between the electric and toroidal dipoles far-field radiation. The electric and magnetic fields generate the propagating spherical waves from the source. Nevertheless, examination of the near-fields zone (See Fig. 2d,f for electric fields, and Fig. 2g,i for magnetic fields) makes the toroidal nature of the source manifest.

### Experiment

**Figure 4:**
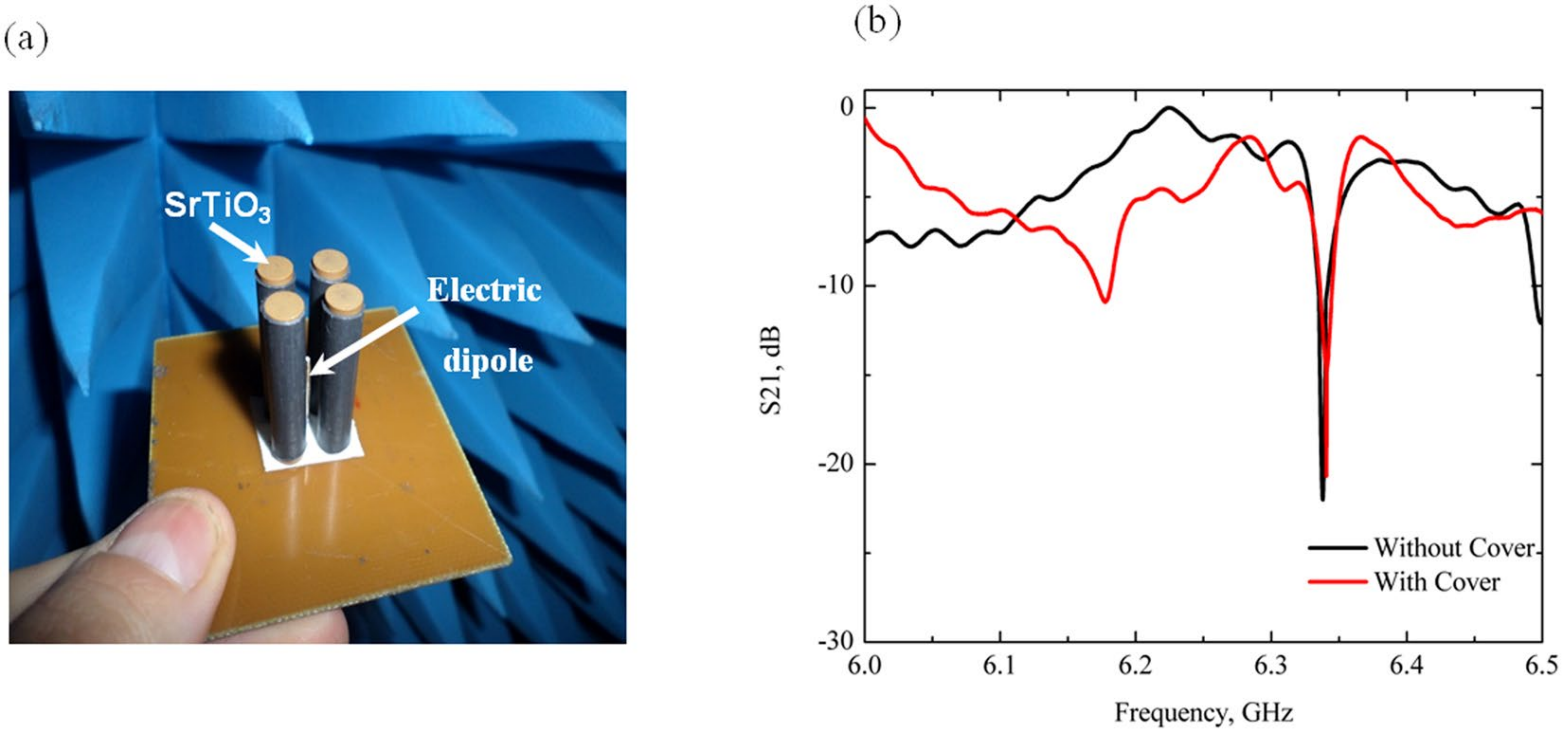
(**а**) Experimental sample of the nontrivial nonradiating all-dielectric anapole based on four- SrTiO_3_ cylinders with diameter 5 mm, excited by electric dipole antenna in the center of meta-molecule. (**b**) The experimental spectra S21 of power received at second antenna relative to the power input to the source: without and with metallic plate on the top of the source, where the black curve corresponds to the case without metallic plate and the red curve is for covered open end of the source by metal plate.

We have also observed the anapole mode experimentally. For this purpose, we constructed the metamolecule excited by the electric dipole antenna placed in the center between the SrTiO_3_ cylinders (Fig. 4a). The electric dipole is connected with the standard coaxial cable. The parameters of the experimental sample are the same as we used in calculations. We placed cylinders on the metallic plate in order to provide symmetrical excitation by the dipole. The size of the plate is 60 mm × 60 mm.

The experimental data shows good qualitative and quantitative agreement with the theoretical predictions. S21 spectra were measured by two antennas with the Vector Network Analyzer Rohde&Schwarz ZVB20 in anechoic chamber as the received signal from the anapole source to the horn receiving antenna located in far-field zone > 4λ (Fig. 4b). The first port is connected with a dipole antenna, while the second one with a horn antenna. We clearly observe a very pronounced deep −25 dB in S21 parameter on the frequency 6.33 GHz that is very close to the theoretical prediction 5.85 GHz (Fig. 1b). The observed discrepancy with the theoretical prediction for S21 parameter is primarily attributed to the deviated values of permittivity and anisotropy of dielectric inclusions. The radiation pattern in the experiment is also omnidirectional in *x*, *y* plane to a high accuracy (Fig. 3a–c). Such a low value of S21-parameter together with the symmetric radiation pattern reveals nonradiating origin of the source in all directions and the cancelation of its far-field radiation.

In order to exclude the radiation in *z*-direction of the cylinders open ends we performed two different experiments. To this end, we covered the open end of the source by a metallic plate. The results (Fig. 4b) clearly indicate that the covering has negligible effect on the anapole mode.

We have also scanned the electric field distribution in the near-zone at the top of the metamolecule by a 2D near-field scanner with a resolution of 0.5 mm. The distribution of electric field at the frequency 6.33 GHz clearly confirms the theoretical prediction (Fig. 2b). Indeed, the near electric field is almost localized within the metamolecule, which is exactly the anapole configuration (Fig. 2b), forming hotspot between cylinders with asymmetry arising due to magnetic Mie-modes in each cylinder. We have also measured the electric field distributions at the frequencies adjacent to the anapole dip. The toroidal radiation at the frequency 6.27 GHz and 6.40 GHz aligns well with the theoretical results (Fig. 2a,c). We point out the far-field radiation of the source beyond the anapole mode frequencies look precisely as the set of spherical waves from an electric dipole (Fig. 2a,c). In the near-field zone, however, the field distributions are sufficiently different and much more close to the toroidal source distribution. For comparison, we added results of simulation and experiment for the purely electric dipole source. Expectedly, that electric dipole radiates omnidirectionally in *x*, *y* plane (Fig. 3d,e).

Let us discuss radiation characteristics of anapole source. Moreover, the anapole mode arises due to destructive interference between the electric and toroidal dipole moments, which have the same far-field radiation patterns. This interference appears only when the dipole moments satisfy a very specific relation **P**=ik**T**. In realistic modelling and even more so in the actual experiment it is not possible to balance the electric and toroidal dipole moments precisely. Still, it is possible to achieve a very strong cancellation. The field maps in the anapole regime (Fig. 2b,e,h) show that the radiation fields indeed are severely suppressed. In contrast, at Fig. 3b the values of RP are normalized. The fact that this radiation pattern is similar to the electric dipoles radiation pattern has a simple explanation. The leading multipole moments of our source near the anapole regime are electric P and toroidal T dipoles. They almost cancel each other near the resonant frequency. However, a very small discrepancy surely remains. If δ**P** = **P** − ik**T** is the value of the mismatch, then what one will see in the far-zone is precisely the radiation field of the electric dipole with value δ**P**.

One can observe that the anapole field distribution at the frequency 6.33 GHz (Fig. 2b) looks more symmetrical in comparison with the toroidal radiation fields on Fig. 2a and c. However, the experiments were performed in an anechoic chamber and the reflection from the chamber walls affects the characteristics of radiating toroidal modes at the same time being imperceptible to the nonradiating anapole mode.

## Discussion

As the summary we discuss possible scenario of anapole nonradiating sources. The papers of the last two years apparently demonstrated the tendency to the anapole excitation in rather simple scatterers such as spherical, cylindrical nano-particles, planar metamolecules and even metallic wires^28^. It is naturally to expect that in simpler structures the anapole can be excited, giving us a chance to look at a new angle on the phenomena concerned with metamaterials and photonics in terms of anapole concept. Due to the nontriviality of anapole sources, the dynamic Aharonov-Bohm effect can be expected, but the caution is needed. In contrast to the static scenario, the dynamic setup involves non-vanishing electric field which also affects the results and hence has to be accurately taken into account. We suppose that the experimental installation would also be more sensitive to various disturbances and hence more demanding that the static experiment. Nevertheless, we believe that the all-dielectric anapole described in this paper is a promising prototype for future nontrivial emitters with the participation of the vector potential and as platform for dynamic Aharonov-Bohm effect.

The anapole sources are interesting as the near-field sensors and cloaked sources/sensors^47, 48^. For example, a subwavelength particle placed near the anapole source will lead to a redistribution between multipoles and destroy the anapole mode leading to observable radiation. As another application we suggest a motion sensor. Displacement of one of the cylinders in various ways (mechanical vibration, acoustics and external laser pumping) amounts to anapole detuning and hence the far-field radiation. Also, since the electric dipole element is under control one can freely switch between the radiating and nonradiating regimes. This is promising for the anapole application as an element of quantum emitters.

All-dielectric anapole source is a remarkable example of the open high Q-factor resonator and modulator. Tunability at the THz and especially the optical range is accompanied by the problem of external access to the closed resonators. The tunability appears here because the cylindrical particles can be manufactured as semiconductors or semiconductor particles can be added to substrate or as extra inclusions. The conductivity of semiconductors can be varied by an external femtosecond pump laser, for instance. The semiconductor inclusion can be placed in the central part of the source where the electric field has its maximum intensity. However, anapole sources or resonators achieving high Q-factor can be strongly tunable with the small pump power needed for this due to low radiating and dissipative losses.

We proposed and theoretically studied a novel class of all-dielectric nonradiating sources exhibiting a resonant anapole response in the microwave part of the spectrum. Our metamolecule is based on subwavelength high-index dielectric cylinders operating in the regime of resonant Mie scattering and excited by electric dipole antenna. We show that the near-field coupling between the individual Mie modes of the cylinders is capable of suppressing all standard multipoles besides the toroidal and electric dipole excitations. The proposed metamolecule can be readily fabricated from low-loss dielectric material SrTiO_3_ and produces a unique field topology at the Mie resonance. Moreover, we demonstrated at the first time radiating/nonradiating nature of toroidal and anapole active modes. Our findings can be useful for future design of nonradiating sources and as a ground for Aharonov-Bohm demonstrations.

## Methods

### Simulations

The electromagnetic properties of the all-dielectric anapole source are computed with the aid of a commercial Maxwell’s equation solver HFSS using the standard transient modeling approach. The simulations also provide the data of electrical currents densities induced in metallic parts of the source and displacement currents in dielectric cylinders, which used to calculate powers radiated of conventional multipoles with taken into account toroidal dipoles^29^.

### Samples fabrication

The all-dielectric anapole source was fabricated from the SrTiO_3_ cylinders placed on a copper plate. The height of cylinders is 35 mm and the radius is 5 mm. Each cylinder consists of 2 cylindrical parts, which are combined and covered by polystyrene grey tubes with permittivity ~2 and thickness 0.5 mm. Due to small permittivity in comparison with SrTiO_3_ cylinders permittivity and small thickness of polystyrene, it does not influence on the proposed effects. The electric dipole of length 10 mm was soldered with SMA connector and fed by coaxial cable.

### Microwave measurements

The experimental spectrum S21 of power received at second antenna relative to the power input to the source was measured in anechoic chamber using Vector Network Analyzer Rohde&Schwarz ZVB20 connected with all-dielectric source and second horn wideband antenna P6–23M. The scanned field distribution was performed by second port of VNA connected with 2D near field scanner with 0.5 mm resolution.

The general discrepancy between experimental and theoretical anapole excitation frequencies arises due to the permittivity of dielectric. In particular, manufacturer declares the permittivity of SrTiO_3_ ~ 37. This value is estimated for dielectric slab and has some deviations depending on frequency, form and quality of the samples. We estimated (See Table 1) in our simulations how the permittivity of dielectric cylinders influences on the resonance frequency.

**Table 1:** One can conclude that the permittivity located close to 35–35.5 well corresponds to our experimental anapole response frequency 6.33 GHz.

f, GHz	6.556	6.4	6	5.85	5.77	5.65	5.49
Permittivity of dielectric	33	35	36	37	38	39	40

Thus, the main discrepancy reason is attributed to the permittivity of dielectrics.
